# Pharmacist and Pharmacy Technician Attitudes and Experiences with Technician-Administered Immunizations

**DOI:** 10.3390/vaccines10081354

**Published:** 2022-08-19

**Authors:** Alexis DiMario, Kenneth Lee McCall, Sara Couture, Wendy Boynton

**Affiliations:** 1 School of Pharmacy, University of New England, Portland, ME 04103, USA; 2 Hannaford Supermarkets & Pharmacy, Scarborough, ME 04074, USA; 3 School of Pharmacy & Pharmaceutical Sciences, Binghamton University, Johnson City, NY 13790, USA

**Keywords:** pharmacy, pharmacy technician, pharmacist, vaccine administration, COVID-19

## Abstract

In response to the increased demand for healthcare services during the COVID-19 pandemic, the Public Readiness and Emergency Preparedness (PREP) Act amendments and guidance authorized pharmacy technicians, who are not otherwise authorized in their state, to administer the Advisory Committee on Immunization Practices (ACIP)-recommended immunizations and COVID-19 vaccines under pharmacist order. Subsequently, many pharmacies nationwide have expanded technician duties to include immunization administration. The primary objective of this study was to evaluate and compare the attitudes and experiences associated with technician-administered immunizations among community pharmacists and technicians. The cross-sectional study evaluated the primary endpoint through the completion of anonymous surveys containing peer-reviewed questionnaires. Pharmacy technicians and their supervising pharmacists were selected to complete the survey at a grocery chain’s pharmacies located in five states across the Northeast if they completed the immunization program and administered at least one immunization. Surveys were drafted using Microsoft Forms and results were analyzed using Microsoft Excel. Chi-squared tests were utilized for comparing categorical variables between groups. A total of 268 survey responses were obtained; 171 responses came from pharmacists and 97 responses came from immunization-certified technicians. Most pharmacists and pharmacy technicians responded that technicians could safely administer vaccines (87.1% and 96.9%, respectively) and competently process and bill vaccine services (90.6% and 99.0%, respectively). In addition, both participant populations responded that technician-administered vaccines improved the workflow of vaccine services (76.6% and 82.5%, respectively) without increasing the likelihood of vaccine errors (56.1% and 78.3%, respectively). When compared with technicians, fewer pharmacists were confident in a technician’s ability to competently prepare vaccines (63.7% vs. 91.8%; *p* < 0.001). A statistically significant association was observed between responses regarding an efficient process for immunizing patients and the likelihood of technician vaccination errors (χ^2^ = 14.36; *p* < 0.01). Pharmacists and pharmacy technicians responded that technicians competently administer immunizations and should participate in more patient-care duties. Multiple states are enacting legislation to include technician vaccine administration as a permanent component of their scope of practice.

## 1. Introduction

On 11 March 2020, the World Health Organization (WHO) declared a global SARS-CoV-2 (COVID-19) pandemic [1]. The novel COVID-19 virus had a dramatic impact on the American healthcare system in 2020 as evidenced by a 9.7% growth in national healthcare spending and demand [1]. There was a drastic increase in the utilization of hospital and clinical services for COVID-19 relief [1]. As a result, elective procedures, general wellness visits, and immunization administration plummeted [1]. In response to the increased demand for healthcare services, the Department of Health and Human Services (HHS) amended the Public Readiness and Emergency Preparedness (PREP) Act to authorize pharmacy technicians, who are not otherwise authorized in their state, to administer the Advisory Committee on Immunization Practices (ACIP)-recommended immunizations and COVID-19 vaccines under pharmacist order during the state of emergency [1,2].

Idaho became the first state to expand the technician scope of practice to include immunization administration in 2017, and Rhode Island and Utah followed shortly thereafter [3,4,5]. Other states have been slow to adopt technician-administered vaccinations into official law as of late 2019 [3,4]. As pharmacies are the most accessible healthcare destination for the public, it is anticipated that the number of vaccination services given by local pharmacies will continue to increase [4]. In the future, more states may incorporate pharmacy technician vaccine administration into law as a gateway to increase vaccination rates and improve access to healthcare [3].

There is limited literature evaluating the overall attitudes and experiences regarding the potential expansion of the pharmacy technician scope of practice to include more patient-care duties such as vaccine administration. A cross-sectional study completed by Ohio Northern University in 2014 found that 93% of pharmacists agreed that pharmacy technicians play a vital role in making the immunization process run smoothly; however, the study failed to evaluate the opinions regarding pharmacy technician immunization administration [4]. Another cross-sectional study conducted in 2018 by the University of Alabama Birmingham found that most pharmacists agreed that pharmacy technicians could competently collect paperwork, process, and bill for vaccines, but only 24% of pharmacists agreed with the idea of technicians administering vaccine doses [6]. 

As for pharmacy technician opinion regarding the willingness to perform emerging tasks in the community setting, a cross-sectional study completed in 2018 found that pharmacy technicians showed a moderate-to-high willingness to perform most emerging tasks; exceptions were vaccine administration and fingerstick blood draw which showed a low willingness [7]. The COVID-19 pandemic started after these studies were conducted and technicians have since been authorized by the PREP Act to administer vaccines under the order of a pharmacist; this may have changed the overall attitudes and opinions regarding the expansion of the pharmacy technician scope of practice to include vaccine administration [1,2]. The primary objective of this cross-sectional study was to evaluate and compare the attitudes and opinions associated with technician-administered immunizations from pharmacists and pharmacy technicians in the community setting.

## 2. Materials and Methods

### 2.1. Study Design and Implementation

This was a cross-sectional study approved by the University of New England IRB committee evaluating the attitudes and experiences of pharmacists and pharmacy technicians regarding technician-administered vaccines. Participants in both groups had to be at least 18 years of age and complete a survey consent form. Pharmacy technicians were selected to complete the survey at a grocery chain’s pharmacies located across 5 states in the Northeast if they completed the 20 h American Pharmacist Association (APhA) immunization certification program and administered at least 1 immunization throughout the course of the pandemic. Their supervising pharmacists were also selected to complete a similar survey. 

The survey was drafted using Microsoft Forms. Two versions of the survey were created to evaluate the primary endpoint: one version was for supervising pharmacists to complete, and the other version was for immunization-certified pharmacy technicians to complete. Both versions of the survey consisted of a 20-item peer-reviewed questionnaire with 7 items inquiring about background information. To ensure that the survey responses remained anonymous, no identifiable information was collected, including employee identification number, email, name, address, or phone number. Participants were asked to rate their level of agreement with each statement on a scale from 1 to 7 with 1 being strongly disagree, 4 being neutral, and 7 being strongly agree. Responses for each survey question that were rated 1–7 were further aggregated into agree, neutral, and disagree for statistical analysis. 

### 2.2. Participant Recruitment

Supervising pharmacists and their immunization-certified technicians were informed about the cross-sectional study analyzing the attitudes and experiences surrounding technician-administered vaccines through a corporate-wide pharmacy email. The email explained the purpose of the study, that participation was voluntary, that responses were anonymous, and how the survey results would be managed, as well as where to find the survey and survey consent form online should one decide to participate. The surveys were launched on 1 February 2022, and participants had 14 days to complete them. A reminder email was sent 6, 8, and 10 days after the survey was launched to help improve participation. All communication was electronic and there was no verbal clarification or information.

### 2.3. Statistical Analysis

Descriptive statistics were calculated to examine pharmacist and pharmacy technician responses regarding technician-administered vaccination by uploading the results from Microsoft Forms into Microsoft Excel. Microsoft Excel was used to organize and compare the survey responses into tables and charts. Chi-squared tests (χ^2^) were utilized for comparing categorical variables between groups (alpha = 0.05). We applied a multivariate logistic regression analysis to the survey responses and reported the adjusted odds ratio and 95% confidence intervals. The dependent variable was agreement with a survey question. The independent variables included title (pharmacist or technician), age (40 years or less versus > 40 years), and years of work experience (10 years or less versus > 10 years). GraphPad Prism version 9.3.0 for Windows was used for the regression analysis.

## 3. Results

### 3.1. Respondent Characteristics

Supervising pharmacists and immunizing pharmacy technicians in a grocery chain’s pharmacies across six states in the Northeast were notified about the study and how to participate through a corporate-wide email. Participants were given 14 days to complete the survey. A total of 268 survey responses were obtained: 171 from pharmacists (63.8%) and 97 from technicians (36.2%). The survey population was majority female (69%) and most were practicing in Maine (36.6%) or New York (29.1%). Respondents were well-distributed across age and pharmacy experience overall (Table 1). However, supervising pharmacists were more likely to be male, older, and more experienced in pharmacy as compared with immunizing technicians (*p* < 0.05). 

### 3.2. Pharmacist Attitudes and Experiences

A total of 171 supervising pharmacists responded to the survey and underwent review as shown in Table 2. The majority of supervising pharmacists agreed that pharmacy technician administration improved the workflow of immunization services in the pharmacy (76.6%) and that technicians could safely administer vaccines (87.1%). Pharmacists also agreed that technicians could competently process and bill for vaccine services (90.6%). In addition, pharmacists reported that technician-administered vaccines helped them focus on pharmacist-related duties (73.7%) and that most patients were comfortable with technicians administering their immunizations (80.1%). Approximately 56.1% of supervising pharmacists responded that the technician scope of practice should expand to include more patient-care duties such as immunization and point-of-care testing. Lastly, most pharmacists agreed that their pharmacy had an efficient process for immunizing patients (87.7%), but only 58.5% of pharmacists agreed that they had sufficient space to immunize patients.

There were some challenges indicated by pharmacists’ survey responses. Many agreed that it was challenging to supervise technician-administered immunizations while performing other duties as a pharmacist (42.7%). In addition, significantly fewer pharmacist survey responses agreed that technicians could competently prepare vaccines as compared with technician responses (63.7% vs. 92%; *p* < 0.001). A total of 21.6% of pharmacists and 10.3% of technicians responded that technician-administered vaccines increased the likelihood of vaccine-related errors. Furthermore, as shown in Table 3, there was a significant inverse association between agreement with an efficient process for immunizing patients and agreement that technician-administered vaccines increase the likelihood of technician vaccination errors (χ^2^ = 14.36; *p* < 0.01).

### 3.3. Pharmacy Technician Attitudes and Experiences

A total of 97 immunizing pharmacy technicians responded to the survey and underwent review as shown in Table 4. Most of the pharmacy technician responses were similar to those of their supervising pharmacists. Most pharmacy technicians agreed that technician-administered immunizations improved the workflow of immunization services in the pharmacy (82.5%). Immunizing technicians also agreed that they could competently process and bill vaccine services (99%), competently prepare vaccines for administration (91.8%), and safely administer vaccines to patients (96.9%) without increasing the likelihood of vaccine errors (78.3%). 

Approximately 69% of technicians agreed that the pharmacy technician scope of practice should expand to include more patient-care duties such as immunizations and point-of-care testing. Technicians, when compared with pharmacists, had higher odds (adjusted for age and years of work experience with multiple logistic regression) of agreement with the statement, “the technician scope of practice should expand to include more patient care duties”: adjusted odds ratio = 1.82 (95% CI, 1.05–3.18). In addition, many responded that patients were comfortable with a technician administering vaccines (86.6%) and that their supervising pharmacists were accessible for questions about immunizations and safety (96.9%). More than half of technician responses showed that administering vaccines did not prevent them from doing other technician duties effectively such as filling, data entry, and third-party billing (51.5%). Lastly, technicians reported that their pharmacy had an efficient process for vaccinating patients (86.6%) and had sufficient space for vaccine administration (67%). 

## 4. Discussion

This cross-sectional study evaluated and compared the attitudes and experiences of supervising pharmacists and immunizing technicians regarding technician-administered vaccines through anonymous surveys. There is limited literature reporting the attitudes and experiences of technician-administered vaccines among pharmacists and technicians. Prior studies have shown that pharmacist survey responses agreed that pharmacy technicians improve the immunization process but none of the studies had an in-depth evaluation of pharmacist and pharmacy technician opinion regarding the expansion of the technician scope of practice to include vaccine administration [4,6,7]. Moreover, these studies were administered prior to the PREP Act which authorized technicians to give vaccines under pharmacist order; consequently, there is a need to re-evaluate the attitudes, experiences, and challenges associated with technician-administered vaccines in the context of the pandemic [2]. 

In addition to the need for access to COVID-19 vaccinations, it is recognized that adult immunization rates for pneumococcal, influenza, and herpes zoster vaccinations did not reach public health targets before 2020 [1,4]. The pharmacy setting has been utilized to administer immunizations for years with success [4,8]. Local pharmacies are one of the most accessible healthcare services with more than 41,000 locations participating in the federal retail pharmacy program for COVID-19 vaccinations [4,8,9]. The demand for vaccine services at pharmacies increased during the pandemic and over 250 million doses of COVID-19 vaccine were administered in that setting [9]. The PREP Act temporarily authorized pharmacy technicians to administer ACIP-recommended immunizations and COVID-19 vaccines under pharmacist supervision during the state of emergency [2]. Prior to the pandemic, Adams et al. insightfully called for regulatory changes to state policy to enable technicians to administer vaccines [5]. Multiple states are now enacting legislation to include technician vaccine administration as a permanent component of their scope of practice [3].

The results of this survey were favorable toward technician-administered vaccinations in the context of the pandemic and support state policies by legislative bodies and boards of licensure which would make this new scope of practice permanent. After training and integrating pharmacy technicians into the immunization workforce, it would be a step back for public health access to rescind their authority to administer vaccines under the supervision of a pharmacist. There were, however, some difficulties noted in this survey, including some pharmacist responses that indicated it was challenging to supervise technicians administering immunizations while performing other duties as a pharmacist. The survey results also noted an inverse relationship between an efficient process for vaccination services and the perceived risk of technician-administered vaccination errors. These survey findings call attention to the need for ensuring adequate staffing and processes to support vaccination services in the pharmacy setting.

This study had several limitations. This was a cross-sectional study that evaluated the primary endpoint through the completion of surveys in which non-response bias was of concern. Furthermore, respondents could have interpreted the statements differently which may have impacted the results. A convenience sample from a grocery chain’s pharmacies located in six states across the Northeast was utilized which limits the generalizability of the results to other settings, states, or countries. Prior to the PREP Act, six states (IA, ID, IN, RI, UT, and WA) allowed pharmacy technicians to administer vaccines [10]. The sample in this study of pharmacists and pharmacy technicians from states in the Northeast may not reflect the attitudes and experiences of pharmacy personnel in states that allowed technicians to administer vaccines before the pandemic. Nonetheless, this study provides insights into pharmacist and pharmacy technician attitudes regarding technician-administered vaccines in the wake of the COVID-19 pandemic.

## 5. Conclusions

Most supervising pharmacists and immunizing technicians surveyed support the recent expansion of the technician scope of practice to include the administration of immunizations. Furthermore, both groups’ survey responses favored the expansion of the pharmacy technician scope of practice to include more patient-care duties under the supervision of the pharmacist. The generalizability of the study findings is limited, and further studies are needed to determine trends among pharmacist and technician attitudes and experiences in the United States.

## Figures and Tables

**Table 1:** Survey respondent characteristics.

	Characteristic	Pharmacist *n* (%)	Technician *n* (%)	Total *n* (%)
Gender ^1^	Male	56 (32.7)	12 (12.4)	68 (25.4)
	Female	105 (61.4)	80 (82.5)	185 (69.0)
	Non-binary	0 (0.0)	2 (2.1)	2 (0.8)
	Unknown	10 (5.8)	3 (3.1)	13 (4.9)
Age (years) ^1^	18–30	34 (19.9)	39 (40.2)	73 (27.2)
	31–40	64 (37.4)	24 (24.7)	88 (32.8)
	41–50	38 (22.2)	17 (17.5)	55 (20.5)
	51–60	29 (17.0)	14 (14.4)	43 (16.0)
	60+	6 (3.5)	3 (3.1)	9 (3.4)
Practicing State	Maine	58 (33.9)	40 (41.2)	98 (36.6)
	Massachusetts	9 (5.3)	9 (9.3)	18 (6.7)
	New Hampshire	24 (14.0)	15 (15.5)	39 (14.6)
	New York	54 (31.6)	24 (24.7)	78 (29.1)
	Vermont	10 (5.8)	7 (7.2)	17 (6.3)
	Multiple States	16 (9.4)	2 (2.1)	18 (6.7)
Work Experience (years) ^1^	0–5	39 (22.8)	36 (37.1)	75 (28.0)
	6–10	30 (17.5)	30 (30.9)	60 (22.4)
	11–20	50 (29.2)	21 (21.6)	71 (26.5)
	20+	52 (30.4)	10 (10.3)	62 (23.1)
Total		171 (100)	97 (100)	268 (100)

^1^ Supervising pharmacists were more likely to be male, older, and more experienced in pharmacy as compared with immunizing technicians (*p* < 0.05).

**Table 2:** Supervising pharmacist survey response result summary (*n* = 171).

Survey Statement	Supervising Pharmacist Response ^1^		
	Agree *n* (%)	Neutral *n* (%)	Disagree *n* (%)
Technicians who have been trained to administer vaccines have improved the workflow of vaccine services in the pharmacy	131 (76.6)	27 (15.8)	13 (7.6)
Pharmacy technicians safely administer vaccines	149 (87.1)	14 (8.2)	8 (4.7)
Pharmacy technicians competently prepare vaccines for administration	109 (63.7)	41 (24.0)	21 (12.3)
Pharmacy technicians competently process vaccine prescriptions including billing	155 (90.6)	4 (2.3)	12 (7.1)
Technician vaccine administration has increased my ability to focus on my duties as a pharmacist	126 (73.7)	20 (11.7)	25 (14.6)
It is challenging to supervise technician vaccine administration while performing my other duties as a pharmacist	73 (42.7)	28 (16.4)	70 (40.9)
Technician vaccine administration increases the likelihood of vaccination errors	37 (21.6)	38 (22.3)	96 (56.1)
The technician scope of practice should expand to include more patient care duties under the supervision of a pharmacist	96 (56.1)	38 (22.3)	37 (21.6)
Only technicians who have the CPhT credential should be trained to administer immunizations	137 (80.1)	13 (7.6)	21 (12.3)
Most patients appear to be comfortable with vaccines administered by a pharmacy technician	187 (80.1)	29 (17.0)	5 (2.9)
Vaccine administration training should be required for technicians who practice in community pharmacy settings	66 (38.6)	34 (19.9)	71 (41.5)
The pharmacy where I work has an efficient process for immunizing patients	150 (87.7)	14 (8.2)	7 (4.1)
The pharmacy where I work has sufficient space to immunize patients	100 (58.5)	21 (12.3)	50 (29.2)

^1^ Responses for each survey question were rated from 1–7 (1 = strongly disagree, 4 = neutral, 7 = strongly agree) and were then aggregated into agree, neutral, and disagree.

**Table 3:** Cross-tabulation showing observed cell counts between pharmacist and pharmacy technician perception of an efficient process or sufficient space for immunization services and the likelihood of vaccination errors associated with technician vaccine administration.

Variable	Response	Technician Vaccine Administration Increases Likelihood of Vaccine Error ^a^			χ^2^ (df)	*p*-Value
		Agree	Neutral	Disagree		
Efficient Process ^b^	Agree	37	37	160	14.36 (2)	<0.01
	Neutral/Disagree	10	12	12		
Sufficient Space ^c^	Agree	28	26	111	2.22 (2)	0.33
	Neutral/Disagree	19	23	61		

^a^ Technician vaccine administration increases the likelihood of vaccine error; ^b^ The pharmacy where I work has an efficient process for immunizing patients; ^c^ The pharmacy where I work has sufficient space for immunizing patients.

**Table 4:** Immunizing pharmacy technician survey response summary (*n* = 97).

Survey Statement	Immunizing Technician Response ^1^		
	Agree *n* (%)	Neutral *n* (%)	Disagree *n* (%)
I can safely administer vaccines	94 (96.9)	1 (1.0)	2 (2.1)
I can competently prepare vaccines for administration	89 (91.8)	6 (6.1)	2 (2.1)
I can competently process vaccine prescriptions including billing	96 (99)	0 (0.0)	1 (1.0)
My supervising pharmacist is accessible when I need to ask questions regarding vaccine administration and safety	94 (96.9)	1 (1.0)	2 (2.1)
Technician administered vaccines have improved the workflow of vaccine services in the pharmacy	80 (82.5)	6 (6.1)	11 (11.4)
Technician vaccine administration increases the likelihood of vaccination errors	10 (10.3)	11 (11.4)	76 (78.3)
Most patients appear to be comfortable with vaccines administered by a pharmacy technician	84 (86.6)	6 (6.1)	7 (7.3)
The work of immunizing patients has prevented me from doing other technician duties effectively (fill, data entry, third party, etc)	31 (32)	16 (16.5)	50 (51.5)
I have adequate training to provide immunization services	91 (93.8)	3 (3.1)	3 (3.1)
Vaccine administration training should be required for technicians who practice in community pharmacy settings	38 (39.2)	24 (24.7)	35 (36.1)
The technician scope of practice should expand to include more patient care duties under the supervision of a pharmacist	67 (69.0)	19 (19.6)	11 (11.4)
The pharmacy where I work has an efficient process for immunizing patients	84 (86.6)	6 (6.1)	7 (7.3)
The pharmacy where I work has sufficient space to immunize patients	65 (67.0)	4 (4.1)	28 (28.9)

^1^ Responses for each survey question that were rated 1–7 (1 = strongly disagree, 4 = neutral, 7 = strongly agree) were further aggregated into agree, neutral, and disagree.

## Data Availability

The data presented in this study are available on request from the corresponding author. The data are not publicly available due to privacy restrictions.
